# Broad host range vectors for expression of proteins with (Twin-) *Strep-*tag*,* His-tag and engineered, export optimized yellow fluorescent protein

**DOI:** 10.1186/1475-2859-12-49

**Published:** 2013-05-20

**Authors:** Thorben Dammeyer, Kenneth N Timmis, Philip Tinnefeld

**Affiliations:** 1 Institut für Physikalische und Theoretische Chemie, NanoBioSciences, Technische Universität Braunschweig, Hans Sommer Str. 10, Braunschweig 38106, Germany; 2 Environmental Microbiology Laboratory, Helmholtz Centre for Infection Research, Inhoffenstr. 7, Braunschweig 38124, Germany; 3 Institut für Mikrobiologie, Technische Universität Braunschweig, Spielmannstr. 7, Braunschweig, 38106, Germany

## Abstract

**Background:**

In current protein research, a limitation still is the production of active recombinant proteins or native protein associations to assess their function. Especially the localization and analysis of protein-complexes or the identification of modifications and small molecule interaction partners by co-purification experiments requires a controllable expression of affinity- and/or fluorescence tagged variants of a protein of interest in its native cellular background. Advantages of periplasmic and/or homologous expressions can frequently not be realized due to a lack of suitable tools. Instead, experiments are often limited to the heterologous production in one of the few well established expression strains.

**Results:**

Here, we introduce a series of new RK2 based broad host range expression plasmids for inducible production of affinity- and fluorescence tagged proteins in the cytoplasm and periplasm of a wide range of Gram negative hosts which are designed to match the recently suggested modular Standard European Vector Architecture and database. The vectors are equipped with a yellow fluorescent protein variant which is engineered to fold and brightly fluoresce in the bacterial periplasm following Sec-mediated export, as shown from fractionation and imaging studies. Expression of *Strep*-tag^®^II and Twin-*Strep*-tag^®^ fusion proteins in *Pseudomonas putida* KT2440 is demonstrated for various ORFs.

**Conclusion:**

The broad host range constructs we have produced enable good and controlled expression of affinity tagged protein variants for single-step purification and qualify for complex co-purification experiments. Periplasmic export variants enable production of affinity tagged proteins and generation of fusion proteins with a novel engineered *Aequorea*-based yellow fluorescent reporter protein variant with activity in the periplasm of the tested Gram-negative model bacteria *Pseudomonas putida* KT2440 and *Escherichia coli* K12 for production, localization or co-localization studies. In addition, the new tools facilitate metabolic engineering and yield assessment for cytoplasmic or periplasmic protein production in a number of different expression hosts when yields in one initially selected are insufficient.

## Background

In the years of exploding genome information, a significant gap of knowledge concerns the function of proteins and information on their diverse modifications and associations. All current efforts in understanding a cell as a system and to *in silico* model and predict pathways and metabolic branch-points, however, rely on the availability of a precise biochemical description not only of the proteins’ kinetic parameters but also on knowledge about localization and essential small molecule and protein interaction partners. The annotated information of a proteins’ specificity and function, even if structural comparison is considered besides sequence homology, is in many cases insufficient [1]. Here, the most common method for biologists and biochemists is the recombinant expression of heterologous proteins in domestic *E.coli* strains. Despite problems of inclusion body formation or low yields for difficult proteins, the produced proteins are in many cases useful for *in vitro* biochemical characterization as well as structural analysis. The vast majority of the microbial diversity however, remains unexplored with regard to experiments involving recombinant production of proteins, due to the limited availability of customized expression tools. This deficit becomes especially aggravating in the emerging era of synthetic and systems biology and is reflected by continuous efforts to develop shuttle plasmids and genetic tools with a broad host range [2-5]. The remaining gap between the gained information on a heterologously expressed protein and its natural behavior can hardly be closed unless a protein can be easily and controllably expressed in the bacteria from which it originates. This is a specific requirement for many experiments like localization and colocalization studies, mutant complementation experiments, chromatin immunoprecipitation or for identification of protein interaction partners.

The analyses of protein interactions, has undergone a rapid evolution in the last years through improvement of affinity- or tandem affinity purifications along with mass spectrometry methods. Here, the Twin-*Strep*-tag^®^ (also known as One-STreP-tag) based application of SPINE (Strep Protein Interaction Experiment) is a prominent example of a simple, successfully developed complex co-purification method [6,7]. The results of high throughput interactome studies often suffer from weak study-to-study result overlap [8] if not intersecting data from different detection methods is extracted [9]. This suggests that careful analyses of individual proteins and protein complexes according to their specific requirements and functional context is a prerequisite for their understanding - a challenge which will be facilitated for many applications with the presented set of tools. Commonly, the placement of an affinity tagged protein under control of the native promoter requires knowledge on conditions under which an expression level suitable for the planned experiment is obtained [10,11]. In our approach, more experimental flexibility is enabled by the use of a controllable promoter and plasmid based systems [12] which is the method of choice for fast and easy selection of suitable expression hosts and culture conditions or generation of several or differentially affinity-tagged proteins. We previously demonstrated the virtue of a plasmid based expression in exploration of new expression hosts for difficult proteins like scFv antibody fragments with *in silico* designed synthetic scFv expression units [13].

Here, we present novel customized plasmids. They combine the advantage of a broad host range, a strong inducible promoter, the latest affinity tags and periplasmic export variants as well as a newly engineered bright yellow fluorescent reporter protein with periplasmic activity, to allow easy localization or one-step purifications of affinity tagged proteins from the periplasm and cytoplasm of Gram-negative bacteria. Furthermore, the new tools contribute towards standardization in vector architecture as they are compatible and stick to the rules of the recently suggested Standard European Vector Architecture (SEVA) format [5]. We demonstrate the functionality of the plasmids in the ubiquitous, metabolically versatile soil bacterium *Pseudomonas putida* KT2440 which provides a genetic and physiological background different from *Escherichia coli* K12 [14]. Besides its considerable potential in a broad range of diverse industrial and environmental applications and the certification as a biosafety strain [15], KT2440 is well known for good expression of heterologous genes [13,16-19] and therefore a valuable alternative production host and interesting candidate for metabolic engineering and systems biological analyses.

## Results and discussion

### Expression plasmid design

**Figure 1:**
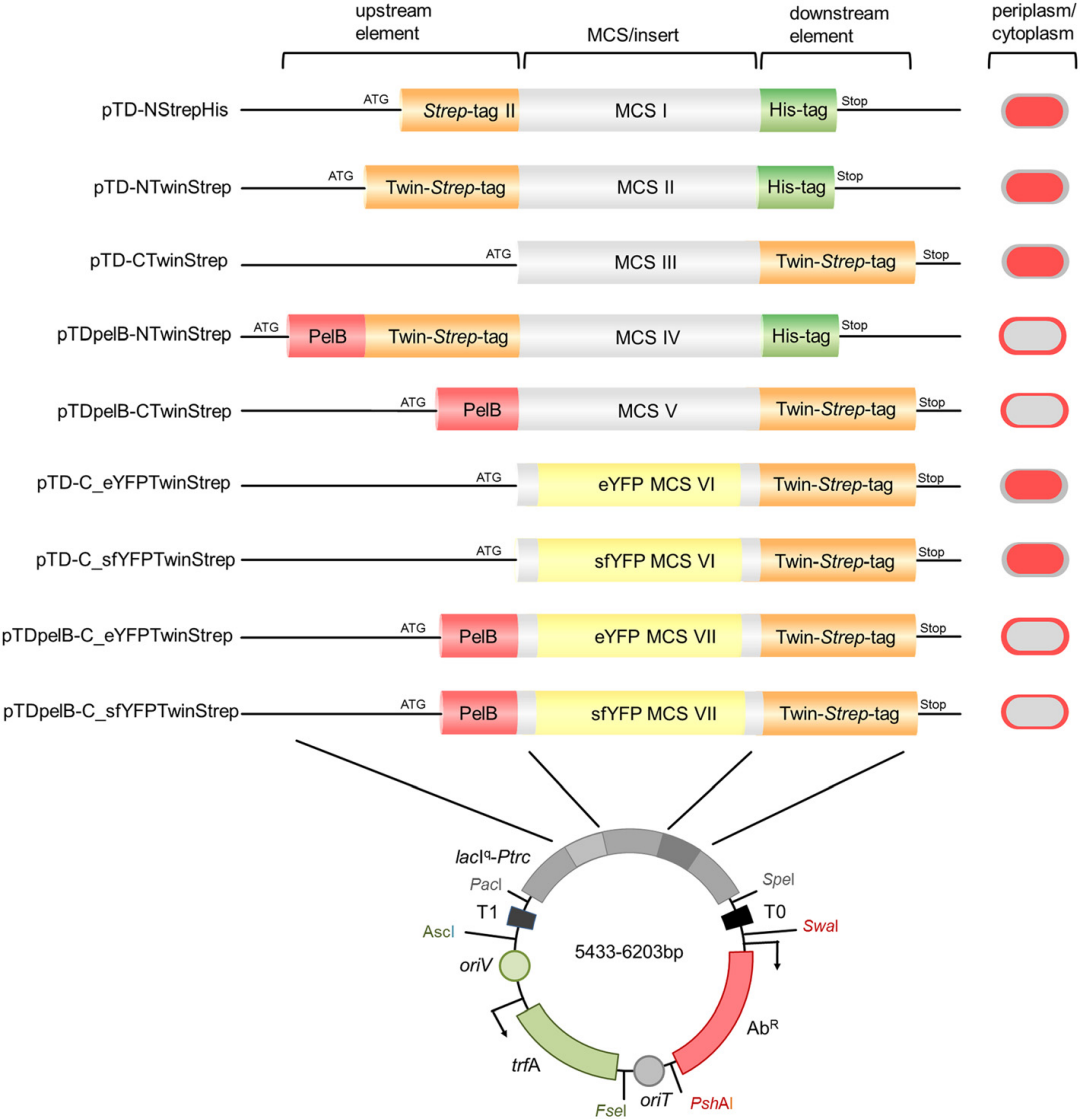
**Schematic representation of pTD-plasmid maps. **Plasmid variants are constructed for periplasmic or cytoplasmic expression and different tag- and reporter gene combinations. Plasmid variants are equipped with the *lacI*^q^-*Ptrc *promoter, the RK2 broad host range origin of replication (oriV/*trfA*) and the streptomycin/spectinomycin (*aadA) *or kanamycin (*aphA*) resistance gene (compare Table 1). Compatibility with SEVA enables exchange of variable modules like replication origin and antibiotic marker *via *the indicated restriction sites, while T0, T1 transcriptional terminators and the conjugation origin oriT are permanent elements.

A novel customized broad host range expression vector cloning system with high application flexibility and versatility is designed to combine well established elements with brand-new features. To achieve a broad host range, we base our system on a plasmid chassis equipped with the well- established RK2 origin of replication [5,20] that has been demonstrated to be efficiently maintained and replicates in more than 30 Gram negative bacteria [5]. Applicability to a large set of hosts is further increased by an oriT origin of transfer which can be used to transfer the plasmids to a host expression strain that is inaccessible to transformation by allowing for conjugative mobilization of the plasmids. A controllable strong inducible promoter is also part of all introduced variants, namely the IPTG inducible *lacI*^q^-*Ptrc* promotor-regulator gene pair. Multiple cloning site (MCS) fragments are optimized, designed entirely *in silico* and chemically synthesized including linkers for cloning *via Mfe*I/*EcoR*I and *Hind*III. The compatible end ligation of *Mfe*I and *EcoR*I cutted sequences thereby ensures retaining of a unique *EcoR*I site for convenient insertion of the sequence of interest (Table 1). The MCS modules comprise ribosome binding sites, transcription starts and unique common restriction sites surrounded by combinations of upstream and downstream elements that are extremely useful in a variety of applications (Figure 1, Additional file 1: Supplemental information 1). Variants allow to generate *Strep*-tag^®^II or Twin-*Strep*-tag^®^ (also known as One-STrEP-tag), tandem *Strep*-tag^®^II fusion proteins. These tags have been well established for single-step affinity purification or mild and rapid purification of intact protein complexes in co-purifications like SPINE (Strep-Protein-Interaction-Experiments) [6,7,21] due to the reversible high specificity binding of the Twin-*Strep*-tag^®^ on immobilized *Strep*-Tactin^®^ and compatibility with formaldehyde crosslinking (see also Additional file 1: Figure S2) and downstream mass spectrometry analyses of proteins [22,23]. In combination with a hexahistidine-tag for immobilized metal affinity chromatography (IMAC) dual-tagging can be achieved and used to control full length production of the protein of interest and/or perform Strep/His tandem affinity purifications.

**Table 1:** Plasmids used and constructed within this study with properties and multiple cloning site characteristics

**Plasmid name**	**Resistance**	**Affinity tags**	**Leader sequence**	**Unique sticky end restriction sites in MCS**
pTD-NStrepHis	Sm	Strep-tagII/His_6_	-	MCSI: *Nhe*I, *Bst*BI, *Eco*RI, *Acc*65I, *Xma*I, *Bam*HI, *Xho*I, *Sal*I, *Nco*I, *Hind*III^1^, *Kpn*I, *Pst*I, *Sac*I
pTD-NTwinStrep_Sm	Sm	TwinStrep/His_6_	-	MCSII: *Eco*RI, *Acc*65I, *Xma*I, *Bam*HI, *Xho*I, *Sal*I, *Nco*I, *Hind*III^1^, *Sac*I *Pst*I, *Kpn*I,
pTD-NTwinStrep_Km	Km	TwinStrep/His_6_	-	MCSII: *Eco*RI, *Acc*65I, *Xma*I, *Bam*HI, *Xho*I, *Sal*I, *Nco*I, *Hind*III^1^, *Pst*I, *Kpn*I, *Sac*I
pTD-CTwinStrep	Sm	TwinStrep	-	MCSIII: *Bam*HI, *Eco*RI, *Sac*I, *Sal*I, *Xba*I, *Xho*I, *Hind*III^2^
pTDpelB-NTwinStrep	Sm	TwinStrep/His_6_	PelB	MCSIV: *Eco*RI, *Acc*65I, *Xma*I, *Bam*HI, *Xho*I, *Sal*I, *Sac*I, *Kpn*I, *Pst*I, *Hind*III^1^
pTDpelB-CTwinStrep	Sm	TwinStrep	PelB	MCSV: *Nco*I, *Eco*RI, *Sac*I, *Xba*I, *Xho*I, *Sac*I, *Kpn*I, *Pst*I, *Hind*III^2^
pTD-C_eYFPTwinStrep	Sm	eYFP-TwinStrep	-	MCSVI: *Bam*HI, *Eco*RI, *Xba*I, *Xho*I, *Hind*III^2^
pTD-C_sfYFPTwinStrep	Sm	sfYFP-TwinStrep	-	MCSVI: *Bam*HI, *Eco*RI, *Xba*I, *Xho*I, *Hind*III^2^
pTDpelB-C_eYFPTwinStrep	Sm	eYFP-TwinStrep	PelB	MCSVII: *Nco*I, *Eco*RI, *Xba*I, *Xho*I, *Hind*III^2^
pTDpelB-C_sfYFPTwinStrep	Sm	sfYFP-TwinStrep	PelB	MCSVII: *Nco*I, *Eco*RI, *Xba*I, *Xho*I, *Hind*III^2^

^1 ^use of this restriction site results in a construct without the C-terminal HIS_6_-tag, addition of a stop codon is recommended; ^2^ use of this restriction site results in a construct without the C-terminal Twin-*Strep*-tag^® ^addition of a stop codon is recommended. Note MCSI, II, IV, contain *Psh*AI sites restricting the SEVA selection marker exchangeability.

Export of proteins to the periplasmic space *via* the general secretory pathway (Sec) is often desirable, either to benefit from the production advantages in the periplasm, like enhanced folding, disulfide bond formation, reduced contaminating background proteins and authentic-N-termini [24], or to address biological functions involving the periplasm, like e.g. cell wall biosynthesis and β-lactam antibiotic function, signaling or transport processes. Universal tools enabling periplasmic production are to our knowledge not available in combination with affinity tags, a broad range origin and inducible promoters. Therefore, we equipped several versions of our multiple cloning sites with the *pelB* signal sequence to allow precise translational fusions with a 22 amino acid periplasmic signal peptide. The PelB signal sequence (ssPelB) has proven to be successful in periplasmic production and precise processing of the pre-proteins of recombinant single-chain scFv antibodies using similar vector chassis in *P*. *putida* KT2440 [13,25].

**Figure 2:**
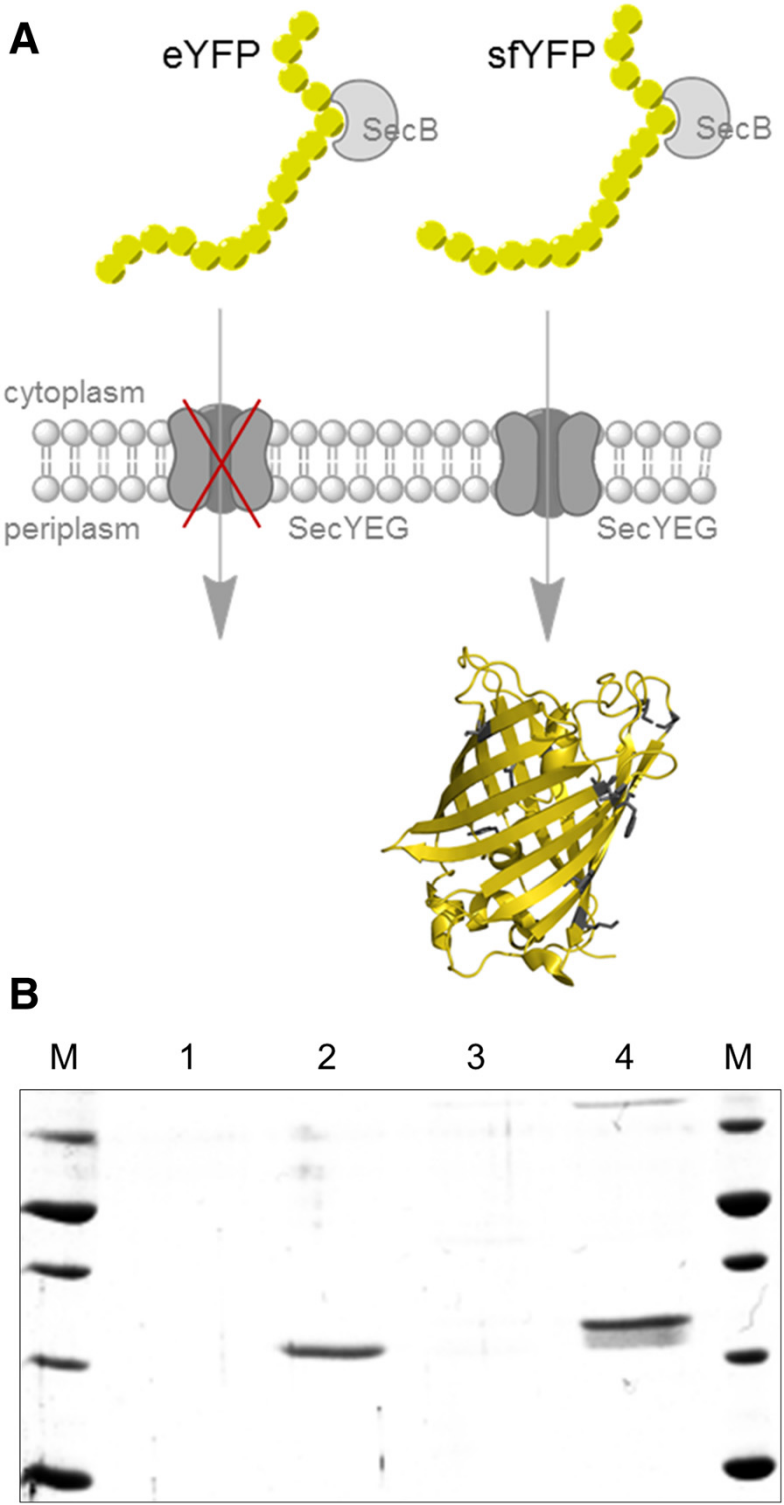
**Yellow fluorescent protein variants and Sec-pathway export. A**) Schematic representation of yellow fluorescent protein variants targeted to the periplasm *via* the Sec-pathway. Standard eYFP targeted to the periplasm does not result in significant levels of protein. In contrast engineered secretion optimized sfYFP yields active fluorescent protein in the bacterial periplasm (cartoon of eYFP generated from pdb 3V3D with mutated residues as grey stick representation. **B**) SDS-PAGE of Strep-tactin affinity purified eYFP (pTDpelB-C_eYFPTwinStrep) (lane 1) and sfYFP (pTDpelB-C_sfYFPTwinStrep) extracted from the periplasm of *E.coli *BL21 (lane 2) and eYFP (pTDpelB-C_eYFPTwinStrep) (lane 3) and sfYFP (pTDpelB-C_sfYFPTwinStrep) extracted from whole cells (lane 4). M = PageRuler Unstained Broad Range Protein Ladder (ThermoScientific) with 70, 50, 40, 30, 20 kDa bands.

To serve as source for enhanced yellow fluorescent protein (eYFP) or for generation of YFP-fusion proteins and reporter gene fusions, reporter gene versions with additional affinity tags were built. While the periplasm promotes disulfide bridge formation, which is a prerequisite for proper folding of many proteins, the popular fluorescent reporter protein eGFP, as its yellow fluorescent counterpart eYFP, are not able to mature in the Gram-negative periplasm following Sec-pathway translocation [26-30], (Figure 2 and Additional file 1: Figure S1). GFP has therefore been proposed as a reporter for cytoplasmic protein localization [28]. To overcome this limitation, we synthetically engineered an additional, periplasmic active YFP variant in this study. These YFP-constructs demonstrate the functionality of the vectors and result in the desired reporter translocation with bright yellow fluorescence in the periplasm of *E.coli* and *P*. *putida* KT2440. Thus, our constructs enable the user to combine the advantages of the periplasmic localization production with the properties of the well folding yellow *Aequorea*-based fluorescent reporter protein.

Moreover, all variants were constructed on a modular basis according to the recently introduced SEVA (Standard European Vector Architecture) platform and database, which enables the principle exchange of three modules, replication module, the promoter MCS module and the antibiotic resistance marker module (Figure 1) with many more exchange modules available [5]. Finally, *Pac*I/*Spe*I bordered modules can be cloned also to pBam1 synthetic Tn5-transposon vector [4,31], allowing for stable chromosomal integration of generated fusion protein expression units.

### Synthetic engineering of periplasmic-export optimized super folder yellow fluorescent protein

For engineering of enhanced (e) and export optimized super folding (sf) YFP expression units, we recruited the established eYFP sequence (pEYFP-C1 Vector, Clonetech, Takara) containing GFP-10C mutations [32,33] as basis. For the eYFP construct, we introduced a A207K dimer interface breaking mutation [34,35] and reverse back-translated the amino acid sequence for optimized *E.coli* translation, erasing restriction sites relevant to the polylinker and chemically synthesized the resulting DNA sequence with ~49,8%GC content (Additional file 1: Supplemental information 1-3) for insertion into the respective constructs (Figure 1, Additional file 1: Supplemental information 1-3). For generation of a bright yellow reporter protein that can cross the cytoplasmic membrane of Gram-negative bacteria *via* the general secretory pathway (Sec) and mature into the proper fluorescent state, we *in silico* designed a second amino acid sequence. Here, we newly combine a series of 10 site directed mutations that have been described to confer beneficial properties with regard to brightness, folding, stability, sensitivity to pH and Cl^-^ concentration to either YFP or GFP. In addition to the A207K exchange, we inserted the cycle3 mutations, F100S, M154T, V164A [36], the enhanced GFP mutation F65L, the folding mutation S176G [33] and the sfGFP mutations S29R, Y40N, N106T, Y146F, I172V [37]. The latter ones are described to confer a super folder phenotype to eGFP which was found to sufficiently enhance folding to produce actively fluorescent sfGFP after Sec-mediated translocation to the oxidizing environment of the Gram negative periplasmic space [26,27]. Notably, the resulting sfYFP has a homology of 96% (228/238 aa) to sfGFP. The engineered sfYFP coding sequence was reverse-back-translated for optimized *P.putida* KT2440 translation. The resulting DNA sequence with a GC content of ~60,8% was freed from intrinsic restriction sites relevant to the polylinker and chemically synthesized with restriction sites for generation of translational fusions with the Twin-*Strep*-tag or ssPelB and Twin-*Strep*-tag in the respective constructs (Figure 1, Additional file 1: Supplemental information 1-3).

### Periplasmic and cytoplasmic expression of eYFP and sfYFP constructs

**Figure 3:**
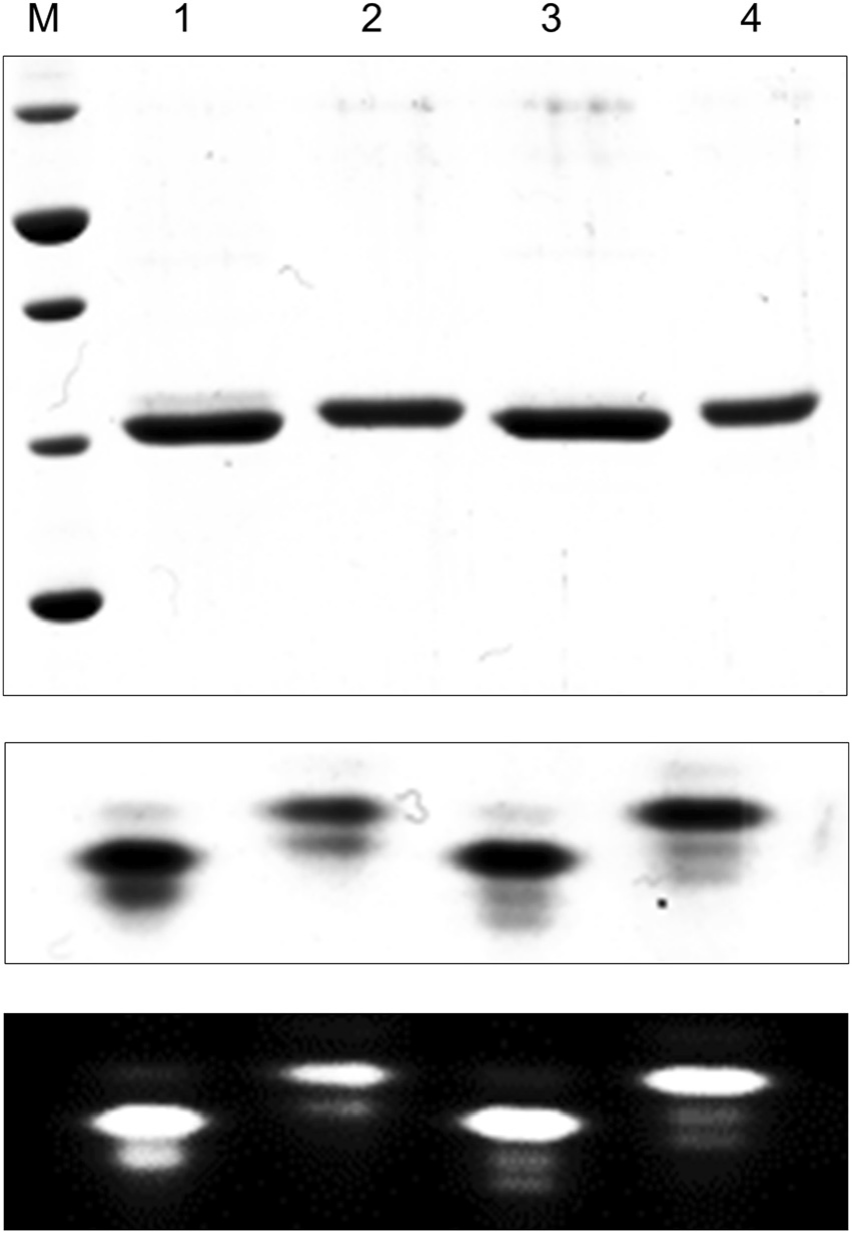
**SDS-and native PAGE of eYFP and sfYFP expressed in *P.putida *KT2440 and *E.coli *Bl21. **Coomassie-stained SDS-PAGE (top), native-PAGE (mid) and native-PAGE fluorescence scan (bottom) of affinity purified 1) eYFP (pTD-C_eYFPTwinStrep); 2) sfYFP (pTD-C_sfYFPTwinStrep) in *E.coli *BL21; 3) eYFP (pTD-C_eYFPTwinStrep) and 4) sfYFP (pTD-C_sfYFPTwinStrep) in *P.putida *KT2440. M = PageRuler Unstained Broad Range Protein Ladder (ThermoScientific) with 70, 50, 40, 30, 20 kDa bands.

To verify and test whether our constructs can be used to express and single step affinity purify eYFP and engineered sfYFP from *E.coli* BL21 (DE3) and *P.putida* KT2440 we transformed the respective constructs pTD-C_eYFPTwinStrep and pTD-C_sfYFPTwinStrep and verified the expression of active fluorescent YFP in the cytoplasm of both reference organisms. Results of SDS-PAGE and native PAGE analyses (Figure 3) show that Twin-Strep-tagged YFP were successfully expressed and single step purified at reasonable high and similar expression levels and are fluorescent. The significant differences in GC content of the YFPs result in no significant differences in expression and fluorescence levels. However, from Figure 3 it can be interpreted that the high GC construct expresses slightly lower protein levels in *E.coli* compared to *P.putida* KT2440 which is in accordance with the previously postulated high plasticity of *P.putida* KT2440 expressing foreign genes with different GC contents [25]. To verify the functionality of our periplasmic PelB-leader sequence expression construct variant and the novel engineered periplasmic fluorescent reporter protein, we transformed vectors pTDpelB-C_TwinStrepeYFP and pTDpelB-C_TwinStrepsfYFP to both reference organisms. Upon whole cell lyses following single step affinity purification as conducted for the cytoplasmic variants we already recognized that none or almost no detectable levels of ssPelB targeted eYFP could be purified from both organisms (Figure 2B, Additional file 1: Figure S1). In contrast we found a significant double band like expression pattern for the Sec-targeted sfYFP variant representing the processed isoform with the 22 amino acid signal peptide cleaved upon translocation and the unprocessed form before translocation, as expected in a phase of continuous translation. Periplasmic extraction resulted in a single band corresponding to the processed isoform, indicating complete or almost complete pre-protein processing as observed for periplasmic scFv antibody production before (Figure 2B) [13].

The results clearly show that Sec targeted eYFP does not yield any significant protein, while the non-targeted eYFP yields similar amounts like our folding engineered YFP in *E.coli* at 37°C as well as in *P.putida* at 30°C. Export of GFP *via* the twin-arginine translocation (Tat) pathway, which translocate proteins in their folded state [38], however is reported [39] indicating that the inactivity of Sec-targeted GFP results from misfolding of the beta-barrel in the periplasm. The two cysteine residues (C49, C71) which do not form a disulfide-bond in the active protein are exposed during folding and might form erroneous intra- or inter molecular disulfide bonds upon translocation to the oxidizing environment of the periplasmic space [26,27]. This might inhibit the proper folding of the barrel structure, which is a prerequisite for the autocatalytic cyclisation and oxidation of the chromophore from the internal tripeptide motive (glycine-tyrosine-glycine) [40,41]. Oligomeric mixed disulfides have been observed upon eukaryotic secretion [26,42] but could not be detected in bacteria [26], which might be a result of insolubility or membrane association. Another reason for the non-detectable unfolded eYFP might be degradation by the Sec-pathway intrinsic quality control machinery. In this regard it has been reported, that Sec targeted, weak folding eGFP variants are degraded by this mechanism, while folding enhanced variants accumulated in the periplasm because they fold too fast and stable to be retained in Sec-secretable, largely unfolded conformation if expressed from high copy plasmids [27,29].

### Fluorescence microscopy of *E.coli* and *P.putida* KT2440 cells expressing a novel, export optimized super-folder YFP

**Figure 4:**
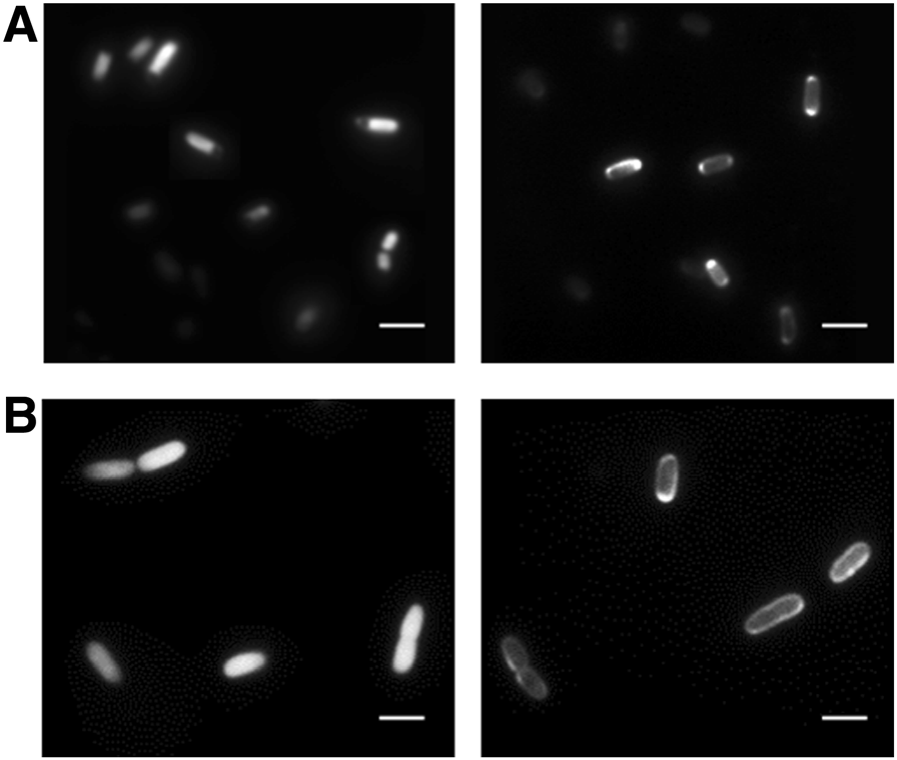
**Fluorescence microscopy images of bacteria expressing export optimized super folder YFP. A**) sfYFP expressed in the cytoplasm (left) and targeted to the periplasm (right) in *E.coli *BL21 and **B**) sfYFP expressed in the cytoplasm (left) and targeted to the periplasm (right) in *P.putida *KT2440 from plasmids pTD-C_sfYFPTwinStrep and pTDpelB-C_sfYFPTwinStrep (scale bar 2.5 μm).

To test whether the novel sfYFP indeed adopts the mature active fluorescent conformation following translocation to the periplasm of Gram negative model bacteria, we conducted fluorescence microscopy. *E*. *coli* BL21 cells expressing sfYFP from vector pTD-C_sfYFPTwinStrep display bright cytoplasmic fluorescence, while the cells expressing Sec targeted sfYFP from vector pTDpelB-C_sfYFPTwinStrep show a clear fluorescence signal localized to and distributed in the periplasmic space of the bacteria (Figure 4A) [30]. Expression of sfYFP from the same broad host range vectors transformed to *P.putida* KT2440 cells display a similar bright cytoplasmic fluorescence for the non-targeted reporter and halo-like peripheral fluorescence signal for the periplasmic expression (Figure 4B).

This results show that the novel combination of 10 site-directed mutations is sufficient to significantly change the phenotype of YFP with regard to protein translocation from the cytosol across the cytoplasmic membrane in bacteria. If unspecific disulfide-bond formation in the oxidizing environment is the main reason for the inactivity of eGFP/eYFP in the periplasm, it is likely that the amino acid mutations improve folding in a manner that reduces the intra- or intermolecular unspecific reactions. It can be speculated that the mutations located ahead of either both or the second of the cysteines are critical for YFP folding while the primary peptide chain emerges from the translocation channel and only the improved folding of the β-barrel enables the intact autocatalytic chromophore formation in the bacterial periplasmic space. In this regard the sfGFP mutations S30R and Y39N have been shown to alter the conformation of the first three β-strands providing the most significant improvements of sfGFPs folding robustness [26,37]. The slightly uneven distribution of sfYFP in the periplasm of *E.coli* (Figure 4A, on the right), which shows a brighter fluorescence signal localized to the cell poles is most likely caused by a previously described reorganizational effect driven by a change in osmolarity caused by resuspension of the cells in phosphate buffered saline during sample preparation [43].

### Controlled expression of homologous cytoplasmic *Pseudomonas putida* KT2440 proteins

To demonstrate the versatility of the novel expression vectors, we further constructed several expression constructs for *P.putida* KT2440 ORFs of different length and functional categories. Control of the expression level could be demonstrated over a wide range on example ORF PP_0479 (RpoA, DNA-directed RNA polymerase subunit alpha) (Additional file 1: Figure S3). Direct comparison of the *Strep*-tag^®^II and Twin-*Strep*-tag^®^ fusion proteins of PP_0479 in single step purifications, resulted in significantly less unspecific background of the Twin-*Strep*-tag^®^ version (Additional file 1: Figure S4, S5) which is also observed for expression of PP_2308 (acyl-CoA thioesterase) (Additional file 1: Figure S5). Expression of dihydrolipoamide dehydrogenase (IpdG) demonstrated the compatibility of method and tag with formaldehyde *in vivo* cross-linking for soluble cytoplasmic proteins (Additional file 1: Figure S2). Further important regulatory RpoN (RNA polymerase factor sigma-54), RpoS (RNA polymerase sigma factor RpoS), NtrC (Nitrogen regulatory protein C), stringent response protein RelA ((p)ppGpp synthetase I SpoT/RelA), and metabolic proteins PhaC1, PhaC2 (poly(3-hydroxyalkanoate) polymerase 1 and 2) and PaaY (phenylacetic acid degradation protein PaaY) could be conveniently produced (Additional file 1: Figures S6-S9). For all tested constructs, expression levels could be detected on SDS-PAGE or anti-*Strep*-tag western blots (Additional file 1: Figure S6). Finally, we tested the utility to perform reproducible co-purification experiments. Here, a similar band pattern of co-purifying proteins could be observed from independent experiments (Additional file 1: Figure S9), indicating the usefulness of our tools for the identification of co-purifying partners in combination with suited mass spectrometry analyses and careful conducted controls.

## Conclusion

We have constructed a new set of modular vectors for the inducible expression of tag- and fluorescent protein fusion proteins compatible with SEVA [5] and demonstrate their applicability for the expression of proteins in the cytoplasm and periplasm of the genetically and metabolically different model bacteria *E.coli* K12 and *P.putida* KT2440. The broad-host range origin allows metabolic engineering of many Gram-negative bacteria, while the affinity tags *Strep*-tag^®^II, Twin-*Strep*-tag^®^ and HIS_6_-tag enable convenient single-step purification and/or detection of produced proteins. The reproducibly controllable expression allows generation of hyper-production strains as well as pull-down or complex-co-purification experiments like SPINE or complementation experiments of transposon mutant libraries [44]. Variants with monomeric YFPs enable production of reporter gene fusion proteins, localization studies or even single-molecule pull-down [35,45] or single-molecule (sub-diffraction) super resolution microscopy [46] experiments, to gain new insights into the spatial organization of the bacterial proteome. Furthermore, we overcome the previous limitations to secret eYFP *via* the Sec pathway to the periplasm by protein engineering. A novel synthetic combination of amino-acid mutations is sufficient to turn eYFP into a periplasmic translocation optimized super-folder YFP which matures into its active fluorescent conformation at 30°C or 37°C respectively in our model organisms as shown in fractionation and imaging experiments. This should grant access to the periplasmic space for new functional analyses and biotechnological production of fluorescence labeled proteins that require export for disulfide-bond formation. Hence, periplasmic production of antibody-fragment-YFP fusions would allow the recombinant production of sensitive, bright-fluorescent detection probes in a single-step.

## Materials and methods

### Construction of inducible broad host range expression plasmids for affinity purification

Expression plasmids pTD-NStrep, pTD-NTwinStrep, pTD-CTwinStrep, pTDpelB-NTwinStrep, pTDpelB-CTwinStrep are constructed based on a RK2 broad host range plasmid chassis, with inducible lacIq/P*trp* promotor and the *aadA* gene from pVLT35 [47] conferring resistance to streptomycin/spectinomycin, that was used successfully for periplasmic production of scFv antibodies in *P*. *putida* KT2440 [13]. pTD-NTwinStrep was also designed in a kanamycin variant bearing the gene *aphA* from pBAM1 [4]. Individual multiple cloning sites are designed *in silico* and inserted *via Mfe*I/*Eco*RI and *Hind*III cloning, enabling a variety of construct designs by use of common unique standard restriction sites (see Table 1; for sequences of the MCS see Additional file 1: Supplemental information 1).

pTDN-StrepHis was constructed based on the MCS from pASKIBA45+ (IBA) attaching a RBS and a C-terminal HIS_6_-tag using primers fw 5′CGTAGCCAATTG TTTAAAAAGGAGATATACAAATGGCTGGAGCC-3′ and rv 5′GACAAGCTT TTTAAATTAGTGATGGTGATGGTGATGCG-3′ with primer overhangs containing *Mfe*I and *Hind*III restriction sites and are cloned *via Mfe*I/*EcoR*I and *Hind*III into pSEVA-RK2-Sm-lac (pSEVA424) [5,13]. The resulting construct enables the production of N-terminal *Strep*-tag^®^II fusion proteins or dual tagging with N-terminal *Strep*-tag^®^II and C-terminal His_6_-tag (MCSI).

For construction of pTD-NTwinStrep and pTD-CTwinStrep synthetic MCS were designed *in silico* to allow precise N-terminal or C-terminal translational fusions with the Twin-*Strep*-tag^®^ (also known as OneSTrEP-tag), a double SA extended *Strep*-tagII (SA-WSHPQFEK) [48] connected by a glycine rich 10 amino acid flexible linker (GGGS)_2_GG. Additionally pTD-NTwinStrep allows for optional dual tagging with an additional C-terminal His_6_-tag. Designed MCSII; IV and V including RBS were synthesized by MWG (Ebersberg) and MCSIII by GENEART (Regensburg) for cloning *via Mfe*I/*EcoR*I and *Hind*III to the plasmid chassis (pSEVA424/224) [5]. For cloning possibilities and unique restriction sites in the MCS see Table 1 and Figure 1.

Additional constructs are designed *in silico* to allow periplasmic translocation *via* the Sec pathway targeted by the 22 amino acid PelB-signal sequence (ssPelB, MKYLLPTAAAGLLLLAAQPAMA) [49]. As the production of scFv antibodies targeted to the periplasmic space of *P.putida* KT2440 and the single-step affinity purification thereof [13] was successful and those plasmids were highly requested, customized versions for C-or N-terminal tagging, pTDpelB-NTwinStrep and pTDpelB-CTwinStrep are designed for convenient handling containing the 66 bp *Erwinia carotovora pelB*-leader sequence for periplasmic export.

Integrity of all constructs was verified by sequencing of the incorporated fragment. Using the sequencing primers pTDF 5′-TGTGTGGAATTGTGAGCGG-3′ and pTDR 5′-ACTTTGTTTTAGGGCGACTG-3′ that bind to positions 4056–4074 and 4641–44660 of pTDN-StrepHis yielding a fragment length of 281 bp for the plasmid without insert.

### Construction of inducible broad host range expression plasmids for fusions to and expression of engineered yellow fluorescent protein

For generation of plasmids pTD-C_eYFPTwinStrep and pTDpelB-C_eYFPTwinStrep we amplified the eYFP variant from a synthetic gene. The chosen eYFP amino acid sequence was codon usage adapted using the *E.coli* best reverse backtranslation function of the EditSeq tool of the Lasergene 7 suite (DNASTAR) and the DNA sequence was synthesized at Eurofins MWG (Ebersberg, Germany). For amplification the forward primer eYFPfw1 *EcoR*I 5′ CGAATTCATCGTTTCTAAAGGTGAAG-3′ and reverse primer eYFPrv1 *Hind*III/*Xba*I 5′AAGCTT TCATTATCTAGATTTGTACAGTTCGTCCATACCCAG-3′ were used to add the respective restriction sites. The product is cloned *via EcoR*I and *Xba*I in pTD-CTwinStrep and pTD-CpelBTwinStrep to give the eYFP constructs. For generation of plasmids pTD-C_sfYFPTwinStrep and pTDpelB-C_sfYFPTwinStrep, we amplified a synthetic sfYFP gene. The amino acid sequence was engineered to contain 10 more site directed mutations besides the A207K [34], the cycle 3 mutations, F100S, M154T, V164A [36], the folding mutations, S176G, F65L [33] and the sfGFP mutations, S29R instead of S31R, Y40N, N106T, Y146F, I172V [37]. The sequence was reverse backtranslated and the DNA sequence, codon usage adaptated to *P*. *putida* KT2440 using JCat [50], was synthesized at Eurofins MWG (Ebersberg, Germany). The PCR-product generated using the primers sfYFPfw1 *Eco*RI 5′-CGAATTCGGTGTCGAAGGGCGAAGAACTG and sfYFPrv1 *Hind*III/*Xba*I 5′ AAGCTTTCATTATCTAGAC TTGTACAGTTCGTCCATGCC was cloned *via Eco*RI and *Xba*I into pTD-CTwinStrep and pTDpelB-CTwinStrep to give the sfYFP constructs.

### Generation of expression constructs for *P.putida* KT2440 ORFs

For generation of expression constructs, we use primers with restriction sites *Eco*RI/*Bam*HI and *Hind*III for cloning into pTD-NStrepHis to generate N-terminal fusions to *Strep*-tag^®^II and primers with *Eco*RI and *Xba*I sites for pTD-CTwinStrep to generate C-terminal fusions with Twin-*Strep*-tag^®^ (Additional file 1: Table S1). *E. coli* strain DH5α, chemicals and enzymes for PCR and cloning were purchased from Fermentas/ThermoScientific (St.Leon-Rot, Germany) and New England Biolabs (Ipswich, MA, USA). Native sequences were amplified using high fidelity proof-reading polymerase (Phusion™) and insertion confirmed by restriction digestion after cloning.

### Transformation of *P. putida* KT2440

Competent cells were prepared using buffer containing 300 mM sucrose as described before [51]. 30 ng of plasmid constructs were used to transform 40 μl of competent *P. putida* KT2440 (DSM 6125) cells by electroporation, which was carried out in prechilled 2 mm cuvettes using a Gene Pulser II with pulse controller plus and capacitance extender plus (Bio-Rad, Hempel Hempstead, UK). Cell:DNA mixes were pulsed at 2.5 kV, 25 μF and 200-500Ω resistance, and subsequently plated on selection medium containing 100 μg/ml of streptomycin and spectinomycin.

### Expression of proteins

An 50 ml overnight liquid culture of *E.coli* BL21(DE3) or *P.putida* KT2440 clone with the respective construct (Table 1) was used to inoculate the production culture 1:100. Production test-cultures were grown in 100/200 ml cultures at 150 rpm at 307°C (*E.coli*) and 370°C (*P.putida*) in Luria Bertani (LB) (5g/l yeast extract, 10g/l tryptone, 5g/l NaCl) containing 50μg/ml streptomycin in baffled Erlenmeyer flasks. In the mid logarithmic phase (at OD_600_ of ~ 0.5-0.6), production was induced by addition of isopropyl-ß-D-thiogalactopyranoside (IPTG) at concentrations ranging between 100 μM and 1 mM. The cells were harvested by centrifugation.

### Periplasmic export and signal peptide cleavage

To obtain periplasmic extracts, harvested cells were resuspended in PE buffer (20% (w/v) sucrose, 50 mM Tris, 1 mM EDTA, pH 8), incubated for 30 min on ice with brief vortexing every 5 minutes, and centrifuged at 20,000 rcf for 30 min. Signal peptide cleavage was assessed by electrophoresis of proteins on 12.5% SDS-PAGE.

### Affinity isolation of proteins and protein associations

Proteins were obtained by whole cell lysis by sonication in sonication buffer (50 mM Tris, 100 mM NaCl, 5 mM MgCl_2_, 0,05% (v/v) Triton X-100, pH 8,0), bacterial protein extraction reagent B-Per (Thermo Fischer scientific) according to the manufacturer’s instructions or sonication in B-Per. Whole cell extracts were centrifuged for 15 min at 15,000 rcf to obtain cell-free extracts which, like periplasmic preparations were applied directly to affinity chromatography columns.

We used NI-NTA (Qiagen), for HIS-tag-based affinity purification, or Strep-Tactin Superflow (IBA) columns for *Strep*-tag^®^ II and Twin-*Strep*-tag^®^ based affinity purification. The washing procedures were carried out according to the manufacturers’ recommendations. Purification and complex-co-purifications were verified by electrophoresis on 12.5% SDS-PAGE and expressed proteins identified by semi-dry transfer to PVDF membranes (Peqlab), and anti-Strep-tag detection using Strep-Tactin alkaline phosphatase (AP) conjugate (IBA, Göttingen, Germany) with chromogenic BCIP (5-Bromo-4-Chloro-3'-Indolyphosphate p-Toluidine Salt), NBT (Nitro-Blue Tetrazolium Chloride) detection (AP Blue Membrane Substrate Solution, Sigma).

### Fluorescence microscopy

Imaging of YFP expressing bacteria was carried out on an Olympus IX-71 microscope applying objective-type total internal reflection fluorescence (TIRF) with an oil-immersion objective (PlanApo N 100×, NA 1.40, Olympus. The laser beam was passed through a clean-up filter (Maxline HC 488/2, AHF Analysentechnik, Tuebingen, Germany) and coupled into the microscope objective by a single-band beamsplitter (zt 488 RDC, AHF Analysentechnik). Fluorescence was spectrally filtered by an emission filter (Brightline HC 531/40, AHF Analysentechnik) and imaged onto an EMCCD camera (Ixon DU-897, Andor Technology, Belfast, Northern Ireland). The effective pixel size was 100 nm. Living bacteria were harvested from an expression culture, washed and immobilized in agarose containing phosphate buffered saline.

## Abbreviations

SEVA: Standard European Vector Architecture; MCS: Multiple cloning site; ORF: Open reading frame; eYFP: Enhanced yellow fluorescent protein; sfGFP: Super-folder green fluorescent protein; sfYFP: Super-folder (export optimized) yellow fluorescent protein; RBS: Ribosome binding site; IPTG: Isopropyl-β-D-thiogalactopyranoside; IMAC: immobilized metal ion affinity chromatography; aa: Amino acid; EDTA: Ethylenediaminetetraacetic acid; PVDF: Polyvinylidene fluoride; AP: Alkaline phosphatase; RPM: Rotations per minute; rcf: Relative centrifugal force; SDS-PAGE: Sodium dodecyl sulfate polyacrylamide gel electrophoresis; PCR: Polymerase chain reaction.

## Competing interests

The authors declare that they have no competing interests.

## Authors' contributions

TD designed and coordinated the study, drafted the manuscript, performed the experiments and analyzed the data. PT and KNT helped to draft the manuscript and to design the study. All authors read and approved the final manuscript.

## Supplementary Material

Additional file 1 Supplemental Information 1-3; Table S1; Figures S1-S9. Click here for file
