# Supplementary material for: Broad host range vectors for expression of proteins with (Twin-) Strep-tag, His-tag and engineered, export optimized yellow fluorescent protein
Source: Microb Cell Fact. 2013 May 20;12:49. doi: 10.1186/1475-2859-12-49 (PMC3680311; doi:10.1186/1475-2859-12-49)
Supplement: Additional file 1 — Supplemental Information 1-3; Table S1; Figures S1-S9. [file 1475-2859-12-49-S1.pdf]

Additional file 1:

Broad host range vectors for expression of proteins with (Twin-) *Strep*-tag, HIS-tag and engineered, export optimized yellow fluorescent protein

Thorben Dammeyer[§,1,2], Kenneth N. Timmis[2,3] and Philip Tinnefeld[1]

[1]Institut für Physikalische und Theoretische Chemie, NanoBioSciences, Technische Universität Braunschweig, Hans Sommer Str. 10, 38106 Braunschweig, Germany

[2]Environmental Microbiology Laboratory, Helmholtz Centre for Infection Research, Inhoffenstr. 7, 38124 Braunschweig, Germany

[3]Institut für Mikrobiologie, Technische Universität Braunschweig, Spielmannstr. 7, 38106 Braunschweig, Germany

[§]Corresponding author

Address correspondence to:

Dr. Thorben Dammeyer

NanoBioSciences

Institut für Physikalische und Theoretische Chemie

Hans Sommer Str. 10

38106 Braunschweig, Germany

Email: T.Dammeyer@tu-braunschweig.de

Supplemental Information 1: DNA sequences of customized, synthetic vector multiple cloning sites.

Restriction sites used for vector construction are in bold; ATG and Stopp codons in italic; the RBS is underlined; Strep-tagII/Twin-Strep-tag is marked in orange; HIS6-tag in green and pelB-sequences in violett. MCS *Eco*RI site is in red. Further information on the used plasmid backbones pSEVA424 (GeneBank # KC847293) and pSEVA224 (GenBank # KC847291) is available on http://seva.cnb.csic.es.

MCSI

cgtagc**caattg**tttaaaggagatatacaa*atg*gctagctggagccacccgcagttcgaaaa
aggcgccgagaccgcggtcccgaattcgagctcggtacccggggatccctcgaggtcgacct
gcaggggaccatggtctcaggcctgagaggatcgcatcaccatcaccAtcac*taa*tttaaa
aagcttgtc

MCSII

cgtagc**caattg**gaaggagatatacaa*atg*gctagcgcttggagccacccgcagttcgagaa
aggtggaggttccggaggtggatcgggaggttcggcgtggagccacccgcagttcgaaaaag
gcgccgagaccgcggtcccgaattcgagctcggtacccggggatccctcgaggtcgacctgc
aggggaccatggtctcaggcctgagaggatcgcatcaccatcaccatcac*taa***aagctt**gt
c

MCSIII

cgtagc**caattg**ttaactttaagaaggagatatacat*atg*gatccgaattcgagctccgtc
gactctagagcggccgcactcgagagcgcttggagccacccgcagttcgagaaaggtggagg
ttccggaggtggatcgggaggtggatcgtggagccacccgcagttcgaaaaa*taatgatga***a**
agcttgtc

MCSIV

cgtagc**caattg**gaaggagatatacaa*atg*aagtacctgctgccgaccgccgccgccggcct
gctgctgctggccgcccagccggccatggccagcgcttggagccacccgcagttcgagaaag
gtggaggttccggaggtggatcgggaggttcggcgtggagccacccgcagttcgaaaaaggc

Gccgagaccgcggtcccgaattcgagctcggtacccggggatccctcgaggtcgacctgcag
gggaccatggtctcaggcctgagaggatcgcatcaccatcaccatcac*taa***aagctt**gtc

MCSV

cgtagc**caattg**ttaactttaagaaggagatatacat*atg*aagtacctgctgccgaccgccg
ccgccggcctgctgctgctggccgcccagccggccatggccgatccgaattcgagctccgtcgactctaga
gcggccgcactcgagagcgcttggagccacccgcagttcgagaaaggtggaggttccggaggtggatc
gggaggttcggcgtggagccacccgcagttcgaaaaa*taatgatga***aagctt**gtc

Supplemental Information 2: Translated amino acid sequence of plasmids pTD_C-eYFP-TwinStrep and pTD_C-sfYFP-TwinStrep. Relevant amino acid residue mutations are colored and numbered relative to the translation start MVSK… (F65L ; S176G; F100S, M154T, V164A; S29R; Y40N; N106T; Y146F; I172V; A207K).

eYFP
MDPNSIVSKGEELFTGVVPILVELDGDVNGHKFSVSGEGEGDATYGKLTLKFICTTGKLPVP
WPTLVTTFGYGLQCFARYPDHMKQHDFFKSAMPEGYVQERTIFFKDDGNYKTRAEVKFEGDT
LVNRIELKGIDFKEDGNILGHKLEYNYNSHNVYIMADKQKNGIKVNFKIRHNIEDGSVQLAD
HYQQNTPIGDGPVLLPDNHYLSYQSKLSKDPNEKRDHMVLLEFVTAAGITLGMDELYKSRAA
ALESAWSHPQFEKGGGSGGGSGGGSWSHPQFEK

super-folder YFP
MDPNSVSKGEELFTGVVPILVELDGDVNGHKFRVSGEGEGDATNGKLTLKFICTTGKLPVPW
PTLVTTLGYGLQCFARYPDHMKQHDFFKSAMPEGYVQERTISFKDDGTYKTRAEVKFEGDTL
VNRIELKGIDFKEDGNILGHKLEYNFNSHNVYITADKQKNGIKANFKIRHNVEDGGVQLADH
YQQNTPIGDGPVLLPDNHYLSYQSKLSKDPNEKRDHMVLLEFVTAAGITLGMDELYKSRAAA
LESAWSHPQFEKGGGSGGGSGGGSWSHPQFEK

Supplemental Information 3: Coding synthetic DNA-sequences from plasmids pTD_C-eYFP-TwinStrep and pTD_C-sfYFP-TwinStrep.

eYFP-TwinStrep (% C+G = 49.88)

atggatccgaattcgatcgtttctaaaggtgaagaactgttcaccggtgttgttccgatcct
ggttgaactggacggtgacgttaacggtcacaaattctctgtttctggtgaaggtgaaggtg
acgctacctacggtaaactgaccctgaaattcatctgcaccaccggtaaactgccggttccg
tggccgaccctggttaccaccttcggttacggtctgcagtgcttcgctcgttacccggacca
catgaaacagcacgacttcttcaaatctgctatgccggaaggttacgttcaggaacgtacca
tcttcttcaaagacgacggtaactacaaaacccgtgctgaagttaaattcgaaggtgacacc
ctggttaaccgtatcgaactgaaaggtatcgacttcaaagaagacggtaacatcctgggtca
caaactggaatacaactacaactctcacaacgtttacatcatggctgacaaacagaaaaacg
gtatcaaagttaacttcaaaatccgtcacaacatcgaagacggttctgttcagctggctgac
cactaccagcagaacaccccgatcggtgacggtccggttctgctgccggacaaccactacct
gtcttaccagtctaaactgtctaaagacccgaacgaaaaacgtgaccacatggttctgctgg
aatttgttaccgctgctggtatcaccctgggtatggacgaactgtacaaatctagagcggcc
gcactcgagagcgcttggagccacccgcagttcgagaaaggtggaggttccggaggtggatc
gggaggtggatcgtggagccacccgcagttcgaaaaa

sfYFP-TwinStrep (% C+G = 60.78)

atggatccgaattcggtgtcgaagggcgaagaactgttcaccggcgtggtgccgatcctggt
ggaactggacggcgacgtgaacggccacaagttccgcgtgtcgggcgaaggcgaaggcgacg
ccaccaacggcaagctgaccctgaagttcatctgcaccaccggcaagctgccggtgccgtgg
ccgaccctggtgaccaccctgggctacggcctgcagtgcttcgcccgctacccggaccacat
gaagcagcacgacttcttcaagtcggccatgccggaaggctacgtgcaggaacgcaccatct
cgttcaaggacgacggcacctacaagacccgcgccgaagtgaagttcgaaggcgacaccctg
gtgaaccgcatcgaactgaagggcatcgacttcaaggaagacggcaacatcctgggccacaa
gctggaatacaacttcaactcgcacaacgtgtacatcaccgccgacaagcagaagaacggca
tcaaggccaacttcaagatccgccacaacgtggaagacggcggcgtgcagctggccgaccac
taccagcagaacaccccgatcggcgacggcccggtgctgctgccggacaaccactacctgtc
gtaccagtcgaagctgtcgaaggacccgaacgaaaagcgcgaccacatggtgctgctggaat
ttgtgaccgccgccggcatcaccctgggcatggacgaactgtacaagtctagagcggccgca
ctcgagagcgcttggagccacccgcagttcgagaaaggtggaggttccggaggtggatcggg
aggtggatcgtggagccacccgcagttcgaaaaa

Supplemental Table:

Table S1: Plasmids and oligonucleotides used to construct expression vectors for *P.putida* KT2440 ORFs of different functional categories. Locus tag numbers from the Pseudomonas genome database and gene names if available with forward and reverse PCR-primer-pairs are given. The respective plasmids and restriction sites *Eco*RI/*Bam*HI/*Hind*III used for cloning are underlined.

Locus tag (gene)	Plasmid	Oligonucletide
PP_0479 (rpoA)	pTD-NStrepHis	5`-c gaattc g ATGCAGATTTCGGTAAATG-3`<br>5`-gcg aagctt TCAGGCGGTCGCCTTGTCG-3`
PP_0479 (rpoA)	pTD-CTwinStrep	5`-c gaattc g ATC CAGATTTCGGTAAATGAGTTC-3`<br>5`-gcg TCTAGA GGCGGTCGCCTTGTCGTCTTTCTTAAG-3`
PP_2308	pTD-NStrepHis	5`-c gaattc g ATGACTTTCAACCAACTGCTCG-3`<br>5`-gcg aagctt TCAGGCGAAAACGGTGACGGTC-3`
PP_2308	pTD-CTwinStrep	5`-c gaattc ATGACTTTCAACCAACTGCTC-3`<br>5`-gcg tctaga GGCGAAAACGGTGACGGTC-3`
PP_5048 (ntrC)	pTD-CTwinStrep	5`-c gaattc g ATcAGCCGAAGTGAAACCGTA-3`<br>5`-gcg tctaga GTGGTCATCACCTTCCTCATC-3`
PP_0952 (rpoN)	pTD-NStrepHis	5`-c gaattc g ATGAAACCATCGCTCGTCC-3`<br>5`-gcg aagctt CTACATCAGTCGCTTGCGTTC-3`
PP_1623 (rpoS)	pTD-NStrepHis	5`-c gaattc g ATGGCTCTCAGTAAAGAAG-3`<br>5`-gcg aagctt CTACTGGAACAATGACTCGCTG-3`
PP_1656 (relA)	pTD-NStrepHis	5`-c gaattc g ATGGTACAGGTGAGAGTGCAC-3`<br>5`-gcg aagctt TCAAGGGGTACGATTACGC-3`
PP_3285 (phaM)	pTD-CTwinStrep	5`- c gaattc g ATcCCTTGCTATCGACTGGAC-3`<br>5`-gcg tctaga TGCGCTTGCCTGGCCCTTGG-3`
PP_5003 (phaA)	pTD-NStrepHis	5`-cagggatccccatATGAGTAACAAGAACAACGATGAGCTAC-3`<br>5`-gcg aagctt TCAACGCTCGTGAACGTAGGTG-3`
PP_5005 (phaC)	pTD-NStrepHis	5`-cagggatccccatATGACAGACAAACCGGCCAAAGGATCG-3`<br>5`-gcg aagctt TCATCGGGTCAGCACGTAGGTG-3`
PP_4187 (IpdG)	pTD-NStrepHis	5`-c gaattc g ATCACCCAGAAATTCGACGTAG-3`<br>5`-gcg aagctt TTAACGCTTCTTACGGTTGG-3`

Supplemental Figures:

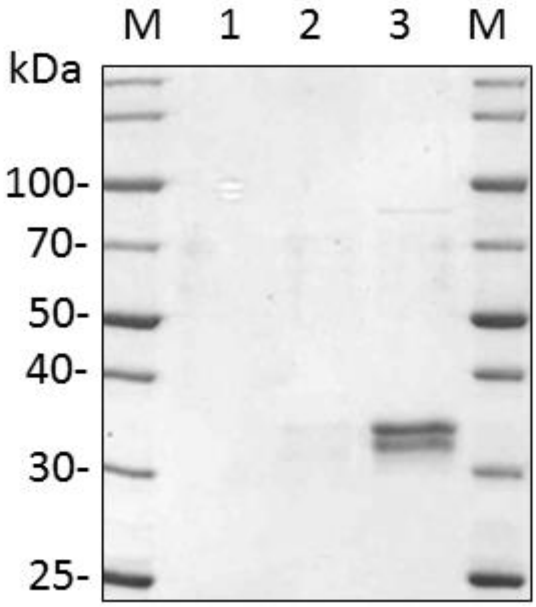

Figure S1: SDS-PAGE of single step-affinity purified eYFP-TwinStrep and sfYFP-TwinStrep. SDS-PAGE of single step-affinity purified eYFP-TwinStrep (2) (from plasmid pTDpelB-C_eYFPTwinStrep) sfYFP-TwinStrep (3) (from plasmid pTDpelB-C_sfYFPTwinStrep) and a similar treated control strain without plasmid (1) from a periplasmic extraction experiment from *P.putida* KT2440. No significant eYFP protein levels can be seen in 2. The lower band in 3 indicates successful production and export of sfYFP. M=PageRuler Unstained Broad Range Protein Ladder (Thermo).

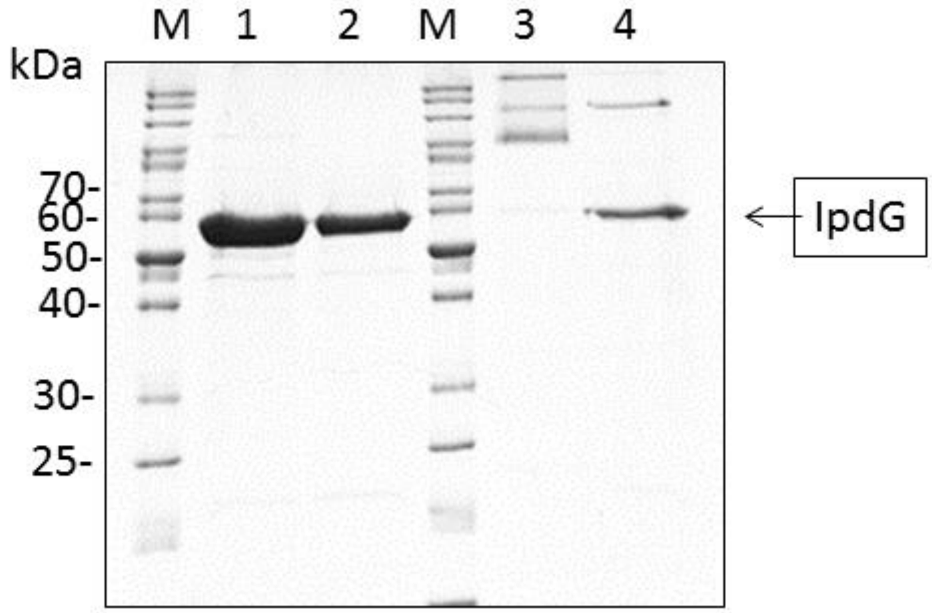

Figure S2: Expression of Strep-PP_4187 (IpdG) in *P.putida* KT2440 and *in vivo* formaldehyd crosslinking. Coomassie stained SDS-PAGE of single step-affinity purified Strep-PP_4187 from plasmid pTD-NStrepHis elution fraction 3 and 4 (1,2) and single step-affinity purification following *in vivo* fromaldehyde crosslinking (3) and reversed crosslinking after 20 min at 95°C in SDS-sample buffer (4). M= PageRuler Unstained Protein Ladder (Thermo).

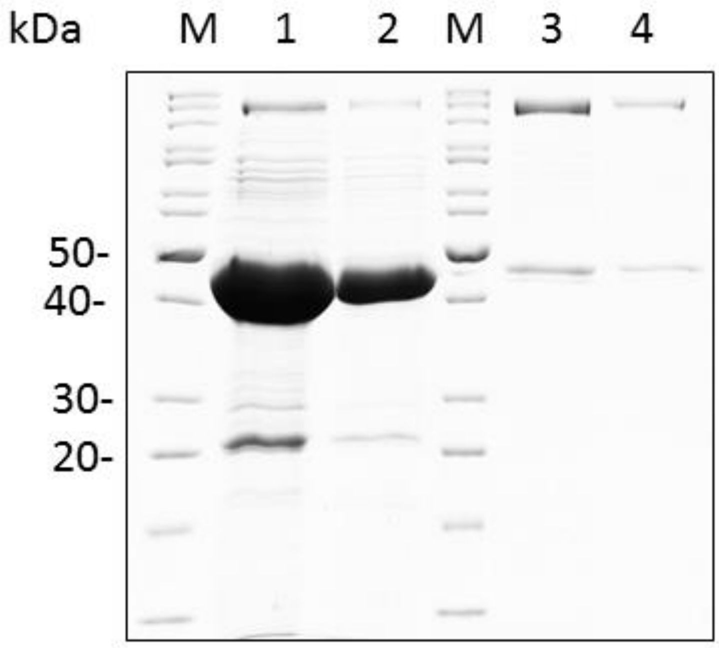

Figure S3: Coomassie stained SDS-PAGE of induced and uninduced cultures of RpoA-TwinStrep from plasmid pTD-CTwinStrep in *P.putida* KT2440. Elution fractions 3+4 after one step purification on Strep-Tactin Superflow of IPTG induced (1, 2) and uninduced (3, 4) cultures. M= PageRuler Unstained Protein Ladder (Thermo).

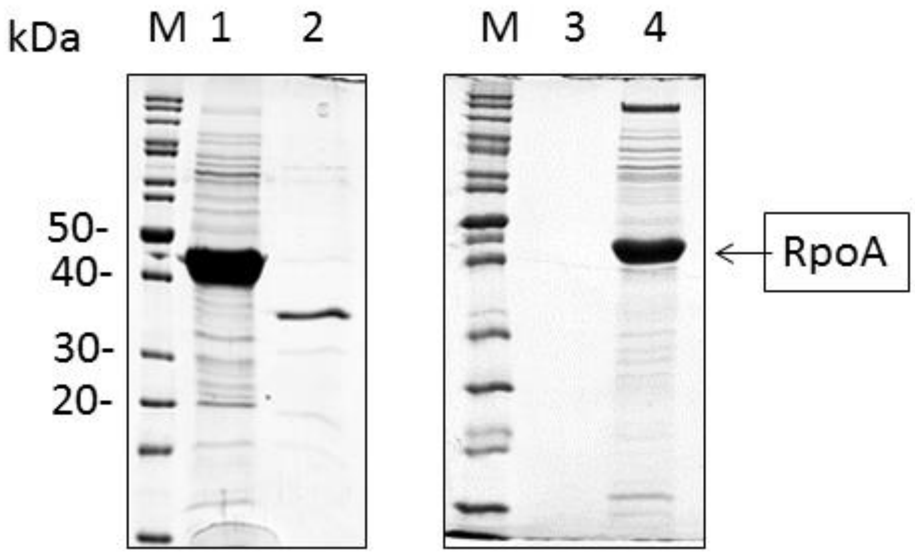

Figure S4: Expression of Strep-RpoA (from pTD-NStrepHis) and RpoA-Twin-Strep from pTD-CTwinStrep in *P.putida* KT2440. Coomassie stained SDS-PAGE after expression and single-step purification of Strep-tagII-RpoA (1) and RpoA-Twin-Strep-tag (4) in comparison to identically treated cultures with the empty plasmid (2, 3). Co-purification experiments with less stringent washing. M= PageRuler Unstained Protein Ladder (Thermo). *Strep*-tag II: SA-WSHPQFEK TwinStrep-tag: SA-WSHPQFEK(GGGS)2GGSAWSHPQFEK.

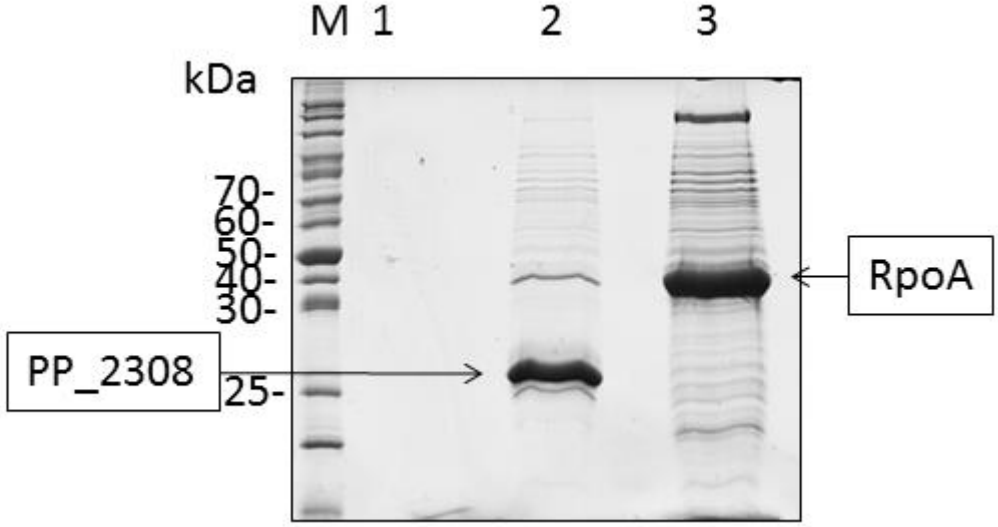

Figure S5: SDS-PAGE of single step-affinity purified PP_2308 and RpoA. Coomassie stained SDS-PAGE of single step-affinity purified PP_2308-TwinStrep (2) and RpoA-TwinStrep (3) from plasmid pTD-CTwinStrep empty plasmid control (1). Co-purification experiments with low expression and less stringent washing. M= PageRuler Unstained Protein Ladder (Thermo).

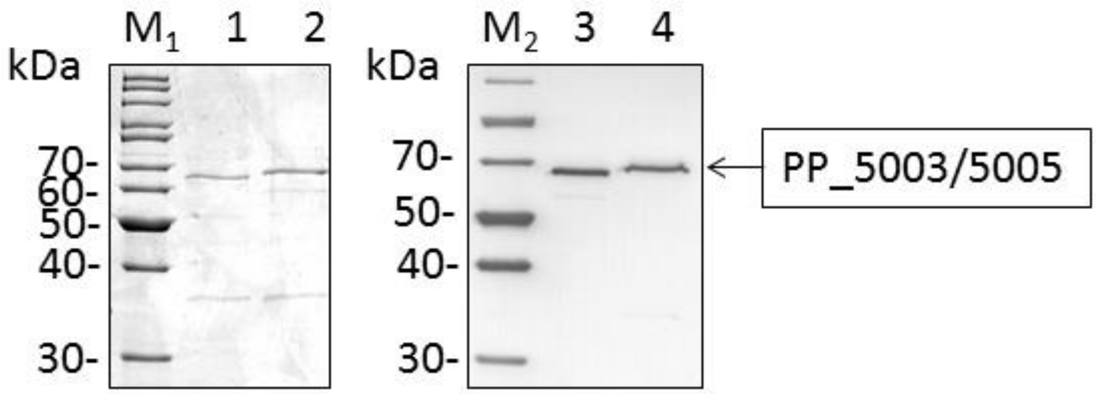

Figure S6: SDS-PAGE and Western-Blot detection of Strep-PP_5003 and Strep-PP_5005. SDS-PAGE of single step-affinity purified (elution fraction 3) Strep-PP_5003 from plasmid pTD-NStrepHis (1) and Strep-PP_5005 (2) and western blot detection with Strep-tactin-AP conjugate (3, 4). M_1 = PageRuler Unstained Protein Ladder (Thermo), M_2 = PageRuler Unstained Broad Range Protein Ladder (Thermo).

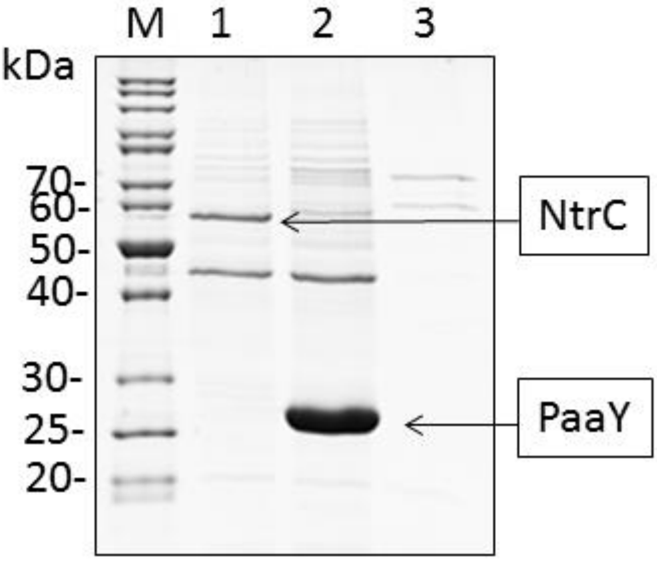

Figure S7: SDS-PAGE of single step-affinity purified PP_5048 and PP_0463. SDS-PAGE of elution fractions 4 of single step-affinity purified PP_5048-TwinStrep (NtrC) (1) and PP_0463-TwinStrep (PaaY) (2) from plasmid pTD-CTwinStrep and empty plasmid control (3) from co-purification experiments with less stringent washing. M= PageRuler Unstained Protein Ladder (Thermo).

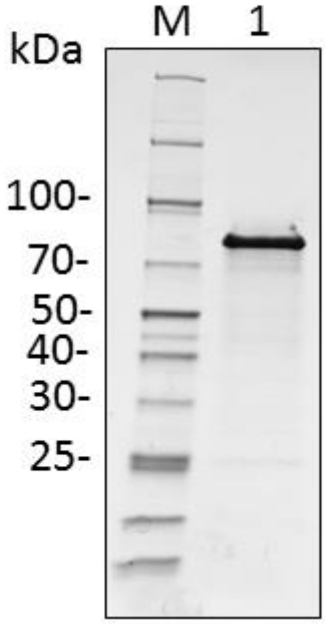

Figure S8: SDS-PAGE of single step-affinity purified PP_1656. SDS-PAGE of single step-affinity purified Strep-PP_1656 (1) from plasmid pTD-NStrepHis. M= PageRuler Unstained Broad Range Protein Ladder (Thermo).

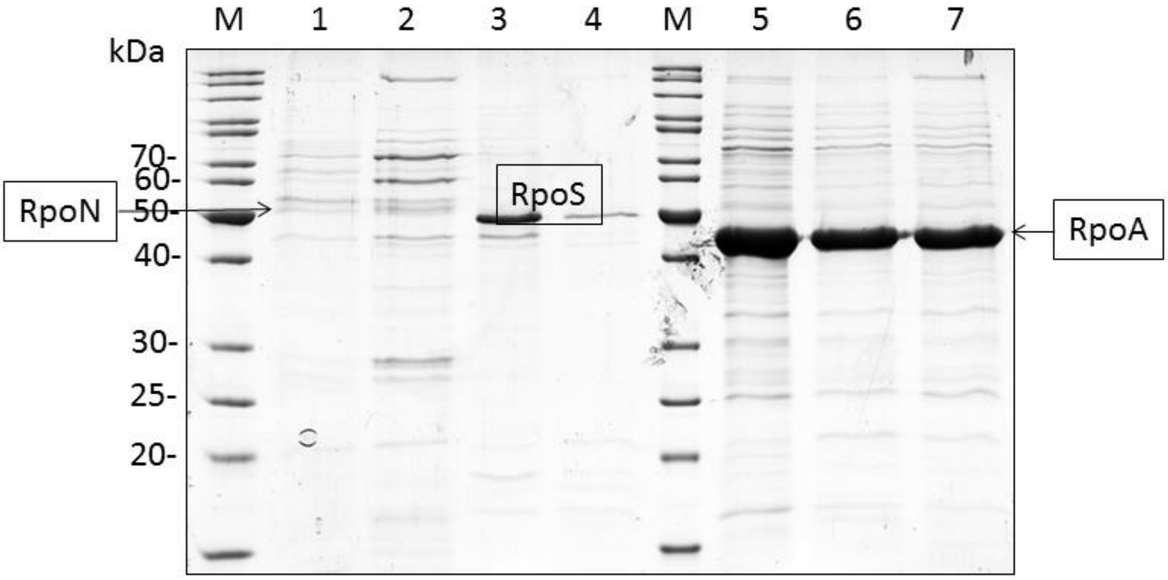

Figure S9: Reproduction of co-purification band-pattern from independent experiments. Independent co-purification experiments with low expression and less stringent washing were done to explore the principle potential applicability in complex co-purification mass -spectrometry experiments for the identification native protein interaction partners. RpoN experiment 1 (1), experiment 2 (2) and RpoS experiment 1 (3) experiment 2 (4) from plasmid pTD-NStrepHis; RpoA experiment 1 elution fraction 3 and 4 (5,6) and experiment 2 (7) from plasmid pTD-CTwinStrep. M= PageRuler Unstained Protein Ladder (Thermo).
